# Prairie Dogs, Persistent Plague, Flocking Fleas, and Pernicious Positive Feedback

**DOI:** 10.3389/fvets.2019.00075

**Published:** 2019-03-28

**Authors:** Dean E. Biggins, David A. Eads

**Affiliations:** ^1^United States Geological Survey, Fort Collins Science Center, Fort Collins, CO, United States; ^2^Department of Biology, Colorado State University, Fort Collins, CO, United States

**Keywords:** plague, flea, *Yersinia pestis*, feedback, epizootic, rodent, enzootic

## Abstract

Plague (caused by the bacterium *Yersinia pestis*) is a deadly flea-borne disease that remains a threat to public health nearly worldwide and is particularly disruptive ecologically where it has been introduced. We review hypotheses regarding maintenance and transmission of *Y. pestis*, emphasizing recent data from North America supporting maintenance by persistent transmission that results in sustained non-epizootic (but variable) rates of mortality in hosts. This maintenance mechanism may facilitate periodic epizootic eruptions “in place” because the need for repeated reinvasion from disjunct sources is eliminated. Resulting explosive outbreaks that spread rapidly in time and space are likely enhanced by synergistic positive feedback (PFB) cycles involving flea vectors, hosts, and the plague bacterium itself. Although PFB has been implied in plague literature for at least 50 years, we propose this mechanism, particularly with regard to flea responses, as central to epizootic plague rather than a phenomenon worthy of just peripheral mention. We also present new data on increases in flea:host ratios resulting from recreational shooting and poisoning as possible triggers for the transition from enzootic maintenance to PFB cycles and epizootic explosions. Although plague outbreaks have received much historic attention, PFB cycles that result in decimation of host populations lead to speculation that epizootic eruptions might not be part of the adaptive evolutionary strategy of *Y. pestis* but might instead be a tolerated intermittent cost of its *modus operandi*. We also speculate that there may be mammal communities where epizootics, as we define them, are rare or absent. Absence of plague epizootics might translate into reduced public health risk but does not necessarily equate to inconsequential ecologic impact.

## Introduction

Plague is a zoonotic disease (caused by the bacterium *Yersinia pestis*) that has a long history of causing human suffering and massive death rates. *Y. pestis* is a generalist vectored by a wide range of fleas (Siphonaptera) (1) and infecting a wide range of mammalian species. The impact of plague on humans has motivated much research, but the complexities caused by the array of hosts and fleas as they interact with each other and their environments have left many ecological questions unanswered (2). Plague has colonized North America, South America, and portions of Africa and southeast Asia, at least, but relatively little attention has been devoted to plague as an invasive disruptor of ecosystems or its effect on species of conservation concern (3–5). Recent studies of plague in the prairie dogs (*Cynomys* spp.) of North America and their critically endangered associated predator, the black-footed ferret (*Mustela nigripes*), have suggested that this disease played a pivotal role in the decline of these mammals and continues to heavily influence conservation activities for them, and associated species (6–8). More than half the species of rodents of conservation concern in North America occur within regions where plague is present (9); perhaps the plight of ferrets and prairie dogs (PDs hereafter) represents a phenomenon that is more common than has been recognized. Thus, broad conservation and public health issues associated with plague make this disease a prime candidate for consideration within a One Health framework (10).

Two questions that are highly relevant to One Health objectives of understanding and managing plague risk are (1) how is plague maintained as a sylvatic disease and (2) what factors lead to epizootic outbreaks? Gage and Kosoy (2, 11) summarized 4 hypotheses for plague maintenance in communities of free-ranging mammals and their fleas: (1) continued enzootic transmission within populations of susceptible hosts and fleas, (2) chronic infection of partially resistant hosts, (3) prolonged survival in fleas, and (4) prolonged survival in soil. Experimental and field evidence has not been able to eliminate any of these hypotheses from consideration, and the 4 ecological mechanisms are not mutually exclusive (2, 11).

Highly susceptible species such as PDs have traditionally escaped notice as potential reservoirs for plague. The logic was, because PDs are “highly vulnerable to plague, they should not be long-term reservoirs of the disease” (12), more dramatically stated as Gunnison's PDs (*C. gunnisoni*) “are clearly not the maintenance species for plague” (13). Historically, partially resistant species were thought to be probable reservoirs or maintenance hosts for *Y. pestis* (14), with microtine or cricetid mice listed as candidates (15–17). Barnes (18) implied that plague was maintained in foothill foci in Colorado, only periodically expanding onto the plains of eastern Colorado and causing epizootics in PDs.

The presumption that PDs and other highly susceptible rodents (19) are not long-term reservoirs of plague implies *Y. pestis* is a periodic invader from residency elsewhere. Recent field studies support the hypothesis of maintenance by susceptible species whose populations often suffer moderate and varying levels of plague-caused mortality during the process and may be periodically decimated by epizootic eruptions. A 5-year controlled study employing flea-control as the treatment to impede plague transmission in 3 PD species implied that there was persistent plague circulation at sub-epizootic levels (20), although vector control effects cannot be unerringly equated to plague effects. In similar studies using vector control, but with experimental plague vaccines added as a second treatment, woodrat (*Neotoma mexicana*) survival in Colorado (2-year study) (Biggins et al. submitted manuscript) and New Mexico (3-year study) (21) was significantly improved by the plague management tools. Unlike vector control, plague vaccine is thought to be specific in its protective effect. In another multi-year study of woodrats (*N. albigula*) in New Mexico, Kosoy et al. (22) collected nest occupancy evidence suggesting maintenance of plague by localized die-offs that shifted over space and time. Finally, either vector control or a plague vaccine improved black-footed ferret survival by > 200% despite lack of epizootic plague during the 4-year experiment in Montana (23). Studies of the genetics of *Y. pestis* and detection of the bacterium during sub-epizootic periods provide additional support for the hypothesis that PDs help to maintain plague or that *Y. pestis* is otherwise maintained locally in or near PD colonies (23–27). New invasions and colonization events may characteristically begin with epizootic plague and later subside into enzootic plague (28) and disease maintenance.

The notion that plague is resident in a geographic area allows for epizootic eruptions in place, without the need for invasion or reinvasion by the bacterium or its resurrection from a quiescent state in soil or elsewhere. Thus, the discussion should be about the scales of eruptions in place vs. movement and the relative importance of each. The parsimonious hypothesis that plague “circulates at much reduced rates among most, if not all, of the same hosts that commonly become infected during epizootics” (2) facilitates a discussion of factors that might promote the transition from enzootic to epizootic transmission rates. One goal in the discussion that follows is to review the roles of flea density, host density, and *Y. pestis* density in that transition, and to propose positive feedback (PFB, hereafter) cycles as definitive elements of epizootic plague. We define PFB as an exponential increase in an effect resulting when the cause is cyclically amplified by the effect such that cause and effect labels become interchangeable. A second goal is to introduce the concept of triggering mechanisms that might initiate runaway PFB.

In addition to the concept of local enzootic plague maintenance by highly susceptible mammalian hosts or their associates, a second influential factor facilitating the PFB cycle might be early phase transmission (EPT) by fleas. Recent evidence on EPT (29–31) is compelling. The speed of the PFB cycle might be dramatically enhanced if infected fleas can immediately transmit *Y. pestis* rather than being delayed 5 days to months while the biofilm-mediated blockage of the proventriculus develops. Also, most fleas die of starvation shortly after becoming fully blocked, ending their ability to contribute to a PFB cycle. These attributes build a strong case for considering EPT as an important contributor to epizootic plague. However, epizootics (as we define them—see below) may last up to several months, thus allowing for blocked fleas to contribute to plague transmission. Another consideration might be the seemingly more efficient transmission reported for blocked fleas (32). Proventricular blockage is not thought to occur in *Oropsylla hirsuta* and *O. tuberculata cynomuris* (33), two important PD fleas, but contradictory results from studies of flea blockage raise questions (32) about the relative involvement of the two forms of transmission in free-ranging rodents.

### Definitions

Before delving into the details of transitions from enzootic plague maintenance to epizootic eruptions, it seems essential to discuss and explicitly define the terms. If plague circulates within a host species at rates that vary along a continuum (2), binomial classification of those rates into epizootic and enzootic is artifactual. Nevertheless, at least two arguments support continued use of these terms. First, the terms and concepts have a long history and, at least at the both ends of the spectrum, convey a sense of real and observable phenomena. When one observes the nearly complete collapse of a PD colony in just a few weeks due to plague, the term epizootic seems intuitively apt. Second, and within the context of this paper, we might give more refined meaning to epizootic if we can associate it with runaway PFB.

Epizootic has been defined as “Pertaining to an epidemic in animals” and epidemic as “a disease affecting a high proportion of the population over a wide area” (34). There is no temporal component to this definition, and the vagueness of “high proportion” and “wide area” render such definitions inadequate for our purposes. Because the definitions might vary somewhat when considering different species and contexts, it is useful for authors to define these terms in each individual report. For example, Biggins et al. (20) described epizootics as resulting in the deaths of >90% of a PD population and enzootic plague as affecting lesser proportions, but they did not provide temporal or spatial criteria. Ramakrishnan (21) used the 90% mortality cutoff but required the episode to occur within 3 months and over at least 10 ha of habitat. In both examples, “affecting” animals is narrowed to considering plague-caused deaths, which seems appropriate given the lethality of plague and the need for a metric that estimates demographic attributes of populations relevant to conservation. For this paper, we adopt the criteria of Ramakrishnan (21) to distinguish between epizootic and enzootic transmission, with further discussion below about the relationship to PFB cycles.

What if an outbreak takes several years to decimate the population of hosts (a phenomenon we have observed)? Under our definition of enzootic plague, populations can either decline or grow over long periods. What about deaths of just a few PDs that comprise a territorial harem polygynous family, or so-called “coterie” occupying a few hectares? At some point on the scale of individual organism to sub-population to population to range-wide distribution of a species we must pick a defining limit for clarity of communication. Clearly, death of an individual PD cannot define an epizootic, nor should we need extinction of a PD species to define it. Coining phrases like “mini-epizootic” or “small-scale epizootic” captures a sense of the mechanism working at small spatial scales but are semantically inarticulate oxymorons because epizootic and epidemic are defined as large scale phenomena.

The term enzootic may be used in a broad context that considers all forms of plague maintenance, not just the transmission of plague at sub-epizootic rates. It can include *Y. pestis* residing in micro-organisms (35), soil (1), or fleas (2, 36). Here, however, we limit our discussion to active enzootic plague transmission. If epizootic defines only one end of a broad spectrum, enzootic must encompass a truly large range of transmission rates and host mortality. The concept of plague maintenance by low rates of transmission dates back almost to the discovery of *Y. pestis* by Yersin in 1894. Low (37) and Elton (38) used the term “smoldering” plague to describe what we might think of as the slow transmission end of the spectrum. That term has been more recently resurrected (39, 40), but it connotes a rather benign manifestation of the disease that does not seem to accurately depict the moderate rates of transmission and mortality that are common and can have substantial impacts on host populations (20, 21, 23, 41).

### PFB Cycle Components of Epizootic Plague

Fleas are a vital component of the PFB cycles discussed herein. An increase in flea parasitism accompanying epizootic plague was observed at least a half century ago when Shchekunova et al. (42) noted “The dying out of the original inhabitants of burrows was accompanied by a migration of fleas onto surviving rodents and onto new settlers. As a result the index of the abundance of fleas on *O. mongolica* here in the beginning of summer amounted to 3.2 and in the autumn—to 8.5…” Pauli et al. (43) uses the term “swarming” of fleas onto hosts during epizootics. Tripp et al. (44) suggests “Concentration of infected fleas on surviving animals may account for the rapid spread of plague during epizootics.” Salkeld et al. (40) mentions that “transmission rates snowball” due to “increased abundance of fleas searching for meals” [see also (45)]. These descriptions seem to infer PFB cycles. The graphics and notes on feedbacks from Ray and Collinge (46), the graphic of Reijniers et al. (47), and the discussion on “vicious circles” of disease transmission by Beldomenico and Begon (48) articulate parts of the PFB cycles we emphasize herein.

Disruptive effects of plague on PD social systems may fortify the flocking of infectious fleas to PD hosts. The presence of kin within PD coteries encourages PDs to remain in coterie territories, affording them fitness benefits such as cooperative predator detection and allogrooming to remove ectoparasites (49). As plague transmission increases and kin disappear, PDs likely inspect vacated burrows (e.g., to entomb dead PDs) and risk acquiring infectious fleas (50). Moreover, as PD coteries become vacated, opportunities for cooperation are diminished or eliminated, and PDs can move among former territories (49, 51), allowing them to acquire and ferry infectious fleas (40).

These two mutually reinforcing PFB loops were encapsulated in a general description by Gage (52):

“The rate of plague transmission by fleas also could be influenced by increased contact rates between infectious vectors and susceptible host individuals, with increased contact resulting in a concomitant increase in secondary infections as the disease spreads from an initial focal infection… Transmission rates also have been suggested to increase during epizootics as a result of infectious fleas becoming more and more concentrated on the decreasing number of surviving hosts…”

**Figure 1:**
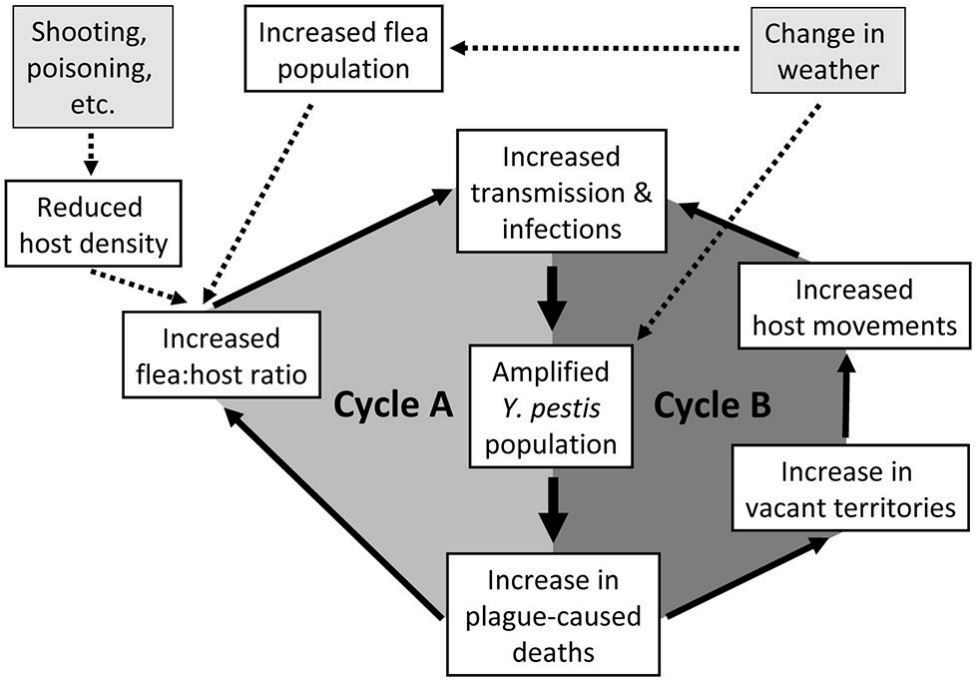
Schematic representation of two synergistic positive feedback cycles involved in epizootic plague eruptions, with emphasis herein on prairie dogs (PDs). Cycle A illustrates an increase in flea:host ratio, and Cycle B illustrates the breakdown of PD territoriality. As the two interconnected loops repeat themselves, and remaining PDs become more mobile, the hazard rate rapidly escalates for each remaining PD. Both loops feed into the transmission rates and ultimately into the plague-caused deaths that the two loops have in common (hence heavier arrows for the central parts of both loops).

We summarize these PFB cycles during an epizootic in PDs (Figure 1) as juxtaposed loops of increasing flea:host ratios (Cycle A) joining increasing host and flea contact due to altered PD social systems and behaviors (Cycle B). The interaction is critical; the 2 loops must be considered together. A triggering event might initiate the primary PFB loop involving altered flea:host ratios. In the short term, the population of plague bacteria rapidly increases, the host population declines, vector numbers remain high, and infections increase. This is soon followed by initiation of the secondary PFB loop as sufficient deaths within coteries cause territorial vacancies that enhance unimpeded PD movements. At that point, both feedback loops operate together to synergistically magnify the overall PFB cycle. As the two connected loops repeat themselves, and remaining PDs become more mobile, the hazard rate rapidly escalates for each remaining PD. Both loops feed into ever higher transmission rates and ultimately into plague-caused deaths that both loops have in common (hence heavier arrows for the central parts of both loops). Triggers are exemplified (Figure 1). A dramatic trigger may initiate an epizootic under less than optimal conditions, or the PFB cycle might spontaneously ignite without any trigger when host and flea densities are high and *Y. pestis* is enzootically abundant in the focal host or associated species.

Weather and habitat conditions doubtless influence hosts, fleas, and *Y. pestis* (53, 54), which we simplistically represent with a single input block (Figure 1). Changes in temperature can influence flea reproduction and survival (55), replication rates of *Y. pestis* (56), and proventricular blockage in fleas (57), thereby influencing transmission rates. Although trophic responses of hosts to weather are likely over longer terms (46), we consider only short-term changes; host populations respond more slowly than populations of fleas or *Y. pestis*. Barnes (18) captured the oversimplification risks of such conceptual models by saying “In this complex and shifting milieu, it is often difficult to determine if fleas or rodents are most important because their roles may change with time, space, and circumstance.” Regardless, an external trigger causing substantial mortality of a subpopulation of hosts, or otherwise optimal conditions for transmission, might initiate an explosive PFB-mediated plague epizootic.

The illustration of the two PFB cycles is representative of early to mid-stages. At some point, there are few PD movements because most PDs are dead, fleas perish from starvation (33), and populations of live *Y. pestis* likely diminish as host carcasses deteriorate or are consumed by scavengers (58, 59). Little is known about demographics and plague-caused mortality of PD fleas (60, 61). However, the primary fleas that seem to be central to *Y. pestis* transmission in PDs (*Oropsylla hirsuta and O. tuberculata cynomuris*) are able to transmit *Y. pestis* before blocking occurs (EPT) (31, 32), are perhaps highly capable of blocked flea transmission (Hinnebusch, personal communication), and might clear some infections but become infected once again when feeding on an infectious host, the latter of which helps to perpetuate plague transmission until the density of hosts is insufficient to support fleas (30, 62).

The combined PFB loops (Figure 1) are described in a temporal context but also have an implied spatial component. As with a metaphoric forest fire PFB cycle, this PFB of fleas and *Y. pestis* cannot erupt for long in one place without running out of PD fuel. It must keep moving. However, unlike fire which moves primarily with the wind, it can move equally well in all directions. In fact, maximizing the area affected per unit of time would involve a feedback cycle that gets triggered in the middle of suitable space, where the movement can be envisioned as expanding circles of impact. This dynamic of *Y. pestis* over time and space may reflect how it maintains itself in an enzootic state (22).

The potentially destructive nature of PFB is commonly illustrated by reference to nuclear weapons. The self-accelerating chain reaction of an atomic bomb releases enormous energy, but the system needs a trigger of conventional explosives (which themselves involve PFB) for activation. Similarly, but at a smaller scale, the bullet from a rifle is propelled down the bore by the self-amplifying explosion of gunpowder, also ignited by a chain of triggering actions. The first of these actions is the shooter physically pulling the rifle's trigger that slams its firing pin into a small, pressure sensitive primer; these actions are analogous to any sudden and localized reduction of PD hosts that increases the flea:host ratio. The primer explodes, triggering the larger PFB explosion in the gunpowder within the cartridge casing and unleashing the destructive power of a speeding bullet, which is analogous to the destructive power of an expanding PFB-powered plague epizootic. Ironically, the rifle and shooter exemplified above could serve as a trigger in our PFB example involving PDs, fleas, and plague.

## Two Field Experiments on Potential PFB Triggers

### The Role of Fleas

Foundational to the PFB hypothesis is the assumption that fleas are critical to plague transmission. Substantial evidence of this has accumulated for >100 years and remains basically unchallenged (2, 11, 32, 36, 58, 63, 64). That said, fleas may not be particularly efficient at transmitting *Y. pestis*, providing an explanation for evolution of high virulence of this pathogen (65); probability of transmission is positively correlated with high levels of host bacteremia that often become lethal. Importantly for the PFB hypothesis, flea inefficiency leads to the need for large numbers of fleas to further increase the probability of transmission and infection (65). Field evidence regarding flea abundance and plague transmission includes flea control experiments that increased rodent survival rates (20, 21) and halted the progression of epizootic plague (66–68). Although less dramatic variation in flea densities may be more difficult to link to plague transmission rates (69), flea parasitism in one study was negatively correlated with PD survival (*Cynomys parvidens*; Eads and Biggins in preparation).

Below, we provide experimental evidence regarding the plausibility of recreational shooting and poisoning of PDs as potential triggers for the flea-plague PFB cycle. Recreational shooting (70) and poisoning (71) are episodic and cause high localized mortality in PD populations. These types of events occur at scales that would seem relevant for PFB triggering. For example, we observed > 97 PDs shot during one morning on a colony of about 300 PDs in Montana (not the colony sampled for study below), and the rodenticide in our South Dakota study was distributed over a 20.6-ha portion of a 70.4-ha colony. Under the PFB hypothesis, episodic host mortality should cause fleas to abandon PD carcasses and flock to living hosts. If so, large numbers of fleas should be collected from burrows near PDs killed by recreational shooters and from burrows in portions of PD colonies that are poisoned.

### Flea Sampling and Data Analyses

On 22 June 2006, we conducted flea sampling in “active” burrows of a black-tailed PD (*Cynomys ludovicianus*) colony in Phillips County, Montana (Colony B-100) on which recreational shooting had occurred within the previous few days (judging from the condition of the PD carcasses found). Burrow activity was classified using the presence of fresh scat (72). Sampling consisted of inserting a plumber's snake tipped with a 15 × 15 cm flannel cloth into each active burrow opening as far as possible for about 30 s and removing the cloth for flea collection and counting (66). The flannel is a crude surrogate for a PD that is investigating the burrow. The insertion technique was done twice at each burrow with a delay between insertions to allow counting and removing fleas from the cloth. Total number of fleas was recorded for each burrow, along with the presence or absence of a dead PD within 1 m of the burrow opening. We graphically presented the data as prevalence (frequencies of burrows from which fleas were collected and not collected), but we used a non-parametric Mann-Whitney test on numbers of fleas collected from each burrow to evaluate the influence of presence or absence of a dead PD at or near the burrow.

Zinc phosphide rodenticide was applied to a portion of a black-tailed PD colony (Cutbank) on the Buffalo Gap National Grassland in South Dakota as part of a “boundary control” effort on 12 December 2017. We sampled active burrows and recorded data as described above, except each burrow (of at least 0.5 m depth) was sampled three times (instead of twice). Sampling was conducted before (5 October) and after application of the rodenticide (13–14 December) on poisoned and non-poisoned portions of the colony. This before-after-control-impact design allowed assessment of treatment effect while controlling for the effect of time, a desirable feature when measuring flea abundance which can vary considerably from month to month (33, 44, 73–76). We evaluated flea abundance using logistic regression models that had time (before or after) and treatment (poisoned or non-poisoned) as predictor variables. A significant (α = 0.05) treatment by time interaction would suggest a treatment-related disproportionate change in fleas over time. Because fleas were much more abundant on this South Dakota colony than on the Montana colony, we used a binomial response variable that considered 6 fleas as the cutoff point (≤6 fleas = 0, > 6 fleas = 1) rather than simple presence or absence (prevalence, as used to graphically illustrate the Montana data).

## Results

**Figure 2:**
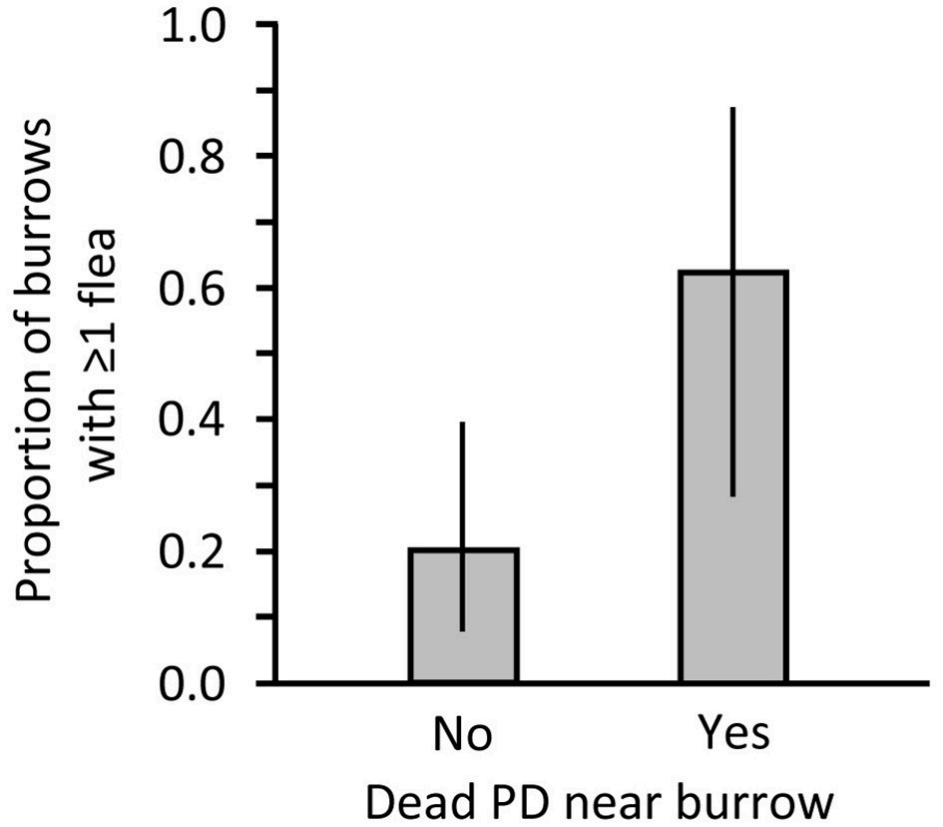
Proportion of burrows on a black-tailed prairie dog (PD) colony in Montana where 1 or more fleas were collected after a recreational shooting event in Montana. Data are presented for burrows at which a dead PD was not (No) or was (Yes) found nearby.

In Montana, we collected 5 fleas from 8 sampled burrows associated with shot PDs and we collected 8 fleas from 25 burrows without a carcass. Average penetration of the sampling apparatus was 2.70 m (range 1–4.5 m). We found 2 additional burrows that contained dead PDs that were visible below the surface. Those 2 burrows were not sampled but suggest there may have been dead PDs present deeper within burrows that were categorized as lacking a PD carcass. Burrow openings accompanied by a dead PD had significantly more fleas than openings without visible carcasses (Mann-Whiney *U* = 52.500, *P* = 0.013) and had higher flea prevalence (Figure 2). Of the 8 burrows with a carcass, 1 had 2 fleas and 1 had 3 fleas; no more than a single flea was collected from any burrow without a carcass.

**Figure 3:**
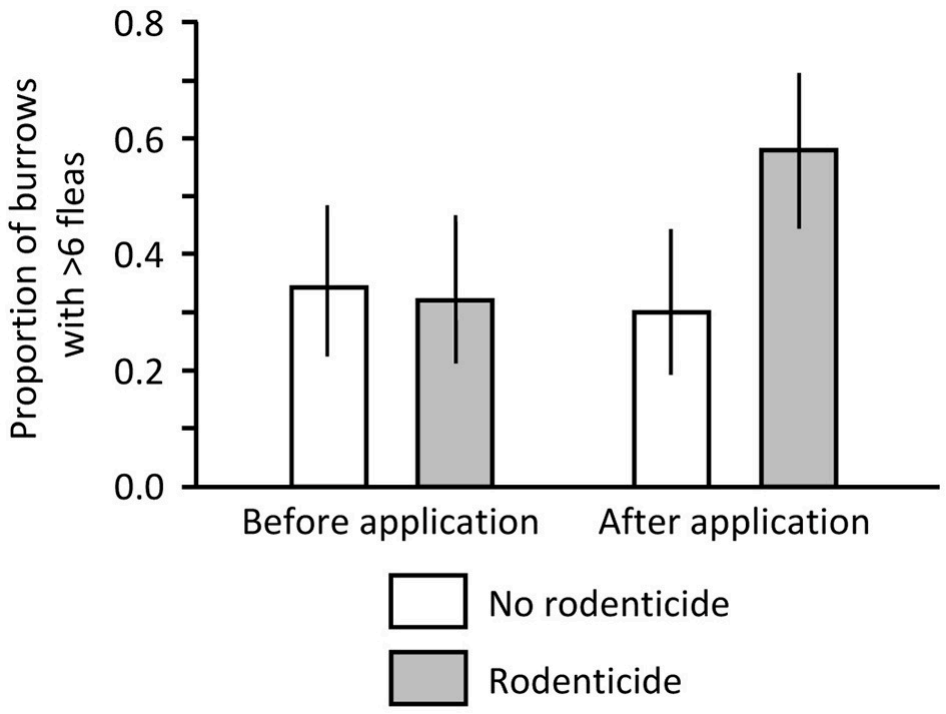
Proportion of burrows on a black-tailed prairie dog (PD) colony in South Dakota where >6 fleas were collected before and after application of zinc phosphide rodenticide.

Before application of zinc phosphide on the South Dakota colony, no dead PDs were found in the non-poisoned or poisoned areas. In contrast, after application, no dead PDs were found in the non-poisoned area but 3 dead PDs and 1 dead PD were found in the poisoned area on 13 and 14 December, respectively. We collected 474 and 390 fleas from 50 swabbed burrows in the non-poisoned and poisoned areas, respectively, before the poisoning event, and we collected 363 and 852 fleas from 50 burrows in non-poisoned and poisoned areas, respectively, after the event. In the logistic regression model, the interaction of time and treatment was significant (Likelihood Ratio *X*^2^ = 4.486, *df* = 1, *P* = 0.034). There was little difference in proportions of burrows with >6 fleas between the poisoned and non-poisoned portions of the colony pre-treatment, but there were nearly twice as many burrows with >6 fleas on the poisoned portion than on the non-poisoned portion following application of the rodenticide (Figure 3; *X*^2^ = 7.955, *df* = 1, *P* = 0.005).

## Discussion

If our inserted flannel swabs were indeed reasonable surrogates for burrow investigations by PDs that explore newly unoccupied territories, they illustrate how flea loads could rapidly increase on PDs due to PFB triggering events (Figure 1). But do flannel swabs provide reasonable indices for flea-host encounters? Perhaps host deaths alter flea behaviors. “During and following an epizootic, fleas migrate to burrow entrances and can be captured in large numbers. When prairie dogs are alive and healthy, fleas tend to remain in the nest where they are not reachable” (18). Questions about detection probabilities (77, 78) within our simple field experiments raise additional uncertainties about measuring flea abundance in burrows. Nevertheless, increased collection of fleas after shooting and poisoning is consistent with the hypothesis that PFB cycles are sometimes triggered by episodic events causing high mortality in a host subpopulation. We might have underestimated the importance of this phenomenon in the poisoning experiment; subsequent observations suggest the zinc phosphide treatment was less effective than expected (causing about 75–80% mortality instead of >90%), meaning the flocking of fleas (which was dramatic) may have been dampened.

### Ramifications of PFB Cycles for Plague Transmission

Intuitively, high host death rates will initiate an increase in flea densities beyond the threshold for epizootic plague (65). Rates of flea collection [e.g., (43, 77)] and flea infection [e.g., (26, 44, 79)] are commonly higher from PD burrows after or during an epizootic than under non-epizootic. Some investigators emphasize the increase in flea abundance and infection as predictors or causes of epizootic plague [e.g., (40, 44, 65)] and others as responses to epizootics [e.g., (42, 63)]. Under the PFB hypothesis, burgeoning flea numbers and infections are both cause and consequence after a cycle begins (Figure 1).

Due to PD social structure and territoriality, flea-plague PFB cycles may occur in a patchy manner (i.e., multiple “explosions” of feedback at the coterie level and slower transmission of *Y. pestis* between coteries). Group deaths within coteries seem likely because coterie members may share burrows as nesting environments (51) and probably share the same sub-population of fleas in their burrows (similar to great gerbils, *Rhombomys opimus*, in Central Asia) (80). After death of the primary coterie defenders, adjustment by members of adjacent coteries is likely (51) and exposure rates of neighbors would be enhanced (40). As epizootic activity increases and plague spreads among coteries, PFB cycles can become self-amplifying until nearly all hosts are parasitized by fleas and succumb to the disease.

Our representation of change in flea:host ratio (Figure 1) as hosts die may be oversimplified due to unequal susceptibility of individuals to flea parasitism and interactions among biotic and abiotic factors. For example, as the flea:host ratio increases during rapid plague transmission, the most susceptible individuals may take the initial brunt of the parasite shift and plague mortality. Adult male PDs could play an especially important role (44); they have much higher flea loads than adult females just after breeding season and often harbor the most fleas in summer and fall (44, 81, 82). Adult male PDs might be the primary initiators of the PRB cycle because of their higher flea loads and because they are the primary coterie defenders (51) and may be the first PDs to explore newly unoccupied territories and thus the first to accumulate newly questing, plague-positive fleas.

Another potential PFB cycle that is intertwined with the flea-plague PFB is mediated by drought. It can be simplistically described as: poor forage (due to drought) leads to water balance and/or energetic limitations (83) that lead to poor host body condition (81) that leads to increased flea loads (74, 81, 84) that lead to even poorer host body condition (55), and so on. This cycle might promote increased circulation of plague due to higher flea loads and perhaps initiate a flea-plague PFB eruption (85). Under sub-optimal conditions for transmission, the developing epizootic may be self-limiting at the point where relatively flea-resistant individuals (e.g., PDs in good body condition) are remaining. Lending support to this hypothesis, Pauli et al. (43) found that PDs surviving an epizootic exhibited improved body condition compared to PDs before the epizootic. In many cases, however, a rapid change in flea:host ratio might overwhelm the entire population, resistant and susceptible alike.

Recreational shooting of PDs might trigger PFB cycles by shifting fleas to the dwindling number of PDs and by affecting PD body condition. During a before-after-control-impact experiment involving shooting of PDs in Wyoming, surviving PDs on shot colonies increased vigilance 8-fold and reduced time spent foraging by 66% relative to PDs on control colonies protected from shooting (86), contributing to a 35% reduction in PD condition. Reductions in PD condition and hypothesized (81) increases in flea parasitism may trigger PFB. Moreover, the significant stresses of recreational shooting (86) may compromise the immune systems of some PDs, causing increases in flea parasitism (55) and mortality in PDs that fail to overwinter, thereby further concentrating fleas on PDs. Although recreational shooting could potentially trigger several types of deleterious PFB cycles, a short-term epizootic cycle (if it developed) would overpower other cycles.

Interactions among a wide array of variables could influence the change in flea:host ratio of the proposed PFB cycles. Flea populations are influenced by many factors that are beyond the scope of detailed discussion here (74, 81, 84, 87–89). Weather and climate at spatial and temporal scales from microsites to El Niño patterns are influential (Figure 1) (90–92) and, as noted above, recent studies suggest precipitation lag effects and host body condition further increase the complexity. Recreational shooting, poisoning, and other sources of host mortality may interact with weather and season. For example, shooting or poisoning after optimal weather conditions for plague transmission may be more likely to trigger an epizootic than shooting or poisoning that follows moisture and temperature conditions that are less favorable for fleas or *Y. pestis*.

Several factors might serve to counter the initiation of epizootic PFB cycles. First, the flea density threshold concept of Lorange et al. (65) is assumed to be critical, although the necessary levels of flea parasitism are unknown for wild, free-ranging hosts such as PDs. Second, intraspecific and interspecific competition among both fleas and hosts could provide negative feedback that impedes the initiation of the flea-plague PFB cycle (55, 93). These phenomena could become interactively complex in systems involving multiple hosts and multiple flea species, but in situations where a single host is primarily responsible for plague circulation, host territoriality could limit transmission to enzootic rates (22). Third, disease transmission rates in general are assumed to be at least somewhat dependent on host densities (94). However, for PDs, it seems that flea densities are more important. An epizootic eruption of plague occurred in Utah PDs when densities (from adjusted visual counts) (72) were just 2.3 PDs ha^−1^ (Biggins unpublished data). Flea parasitism was an important predictor of Utah PD annual survival during a 4-year study; epizootic plague was suspected in many cases, despite low PD densities (Eads and Biggins unpublished data). Thus, it is unsurprising that large rodent control campaigns have failed to eradicate plague in sylvatic systems and that tactic has been abandoned in Russia (95). More localized control of peridomestic rodents, however, can reduce risk of plague exposure in humans (96). We emphasize highly plague-susceptible North American PDs in this treatise, but other species with proportions of populations that are immune to plague would be expected to exhibit much different population dynamics when challenged by plague.

In keeping with the idea that any significant cause of mortality might initiate a PFB cycle (increasing ectoparasite:host ratios), other vector-borne diseases (e.g., tularemia) should also be considered. Triggers might result in secondary interactions between diseases, transforming diseases that might characteristically have a moderate effect (which probably include some diseases native to North America, like tularemia) into triggers for the flea-plague PFB cycle. Conversely, we might consider that plague, operating within its own PFB cycle, might exacerbate the effect of native disease by altering the parasite:host ratio.

### PFB Cycles, Balancing Negative Feedback, and Source-Sink Dynamics

There are examples of PFB that build and sustain ecological systems (97, 98). Nevertheless, “Positive feedback mechanisms are usually associated with instability in a system” (99) and are often considered to be destabilizing and deleterious. Examples are the self-reinforcing nitrogen dynamics of invasive cheat grass (*Bromus tectorum*) in the western U.S (100), the human-triggered algal and microbial feedback loops that threaten coral reefs (101), and even the postulated runaway greenhouse involved in the massive Permian extinction (102). PFB can be facilitative or disruptive (34), depending in part on the status of a process over time, and on the scale of assessment. Taken alone, a PFB seems to be ultimately destructive, but working in concert with negative feedback and other complex interactions, it can contribute to overall stability (103).

Not all PFB cycles are destructive in PDs. One of the more interesting aspects of these tradeoff phenomena in PDs is the balancing of negative feedback and PFB cycles prior to invasion of plague. Over much longer time spans than those for the flea-plague PFB of epizootics, PFB has been discussed in PDs in the context of Allee effect (a positive correlation between population density and average individual fitness) resulting from increased effectiveness of predator warning communications and higher individual survival rates at higher population densities (104). PDs clip grasses and forbs seasonally to maintain unrestricted vision, and repeated clipping of shrubs results in declining shrub densities over periods of years to decades; increased PD densities facilitate this PFB loop (105). In addition to the increased survival rates accompanying this PFB, PDs might have higher birth rates at higher population densities (106). Historically, the slow process of PFB in shrub reduction and increasingly efficient anti-predator behaviors with PD population growth may have gradually come into balance with the negative feedback of coterie territoriality and limiting resources. However, the plague-flea PFB cycle is explosive, and runaway flea-plague PFB will curb other processes.

Plague epizootics may occur in multispecies communities of hosts because *Y. pestis* is a generalist parasite. It might be a mistake to single out a particular host species as the driver of these phenomena, although outbreaks are characteristic in various species of ground squirrels (including PDs). Even within the *Cynomys* genus, manifestations of plague epizootics appear to vary among species. White-tailed PDs (*C. leucurus*) and other species within the *Leucocrossuromys* subgenus may no longer reach peak densities in preferred habitat (grass-dominated sites with few shrubs) because epizootics repeatedly decimate populations that reach reasonable densities (107). From a source-sink perspective, the source has become the poor, shrub-dominated, habitats that maintain enzootic plague, which may have been considered the sinks for these PDs historically. An indirect effect of this phenomenon is failure of white-tailed PDs to create optimum habitat by clipping shrubs and killing them. This is not a true source-sink reversal. A source (prime PD grassland patch) can become a sink because of plague, but the sink (in this case shrubby habitat) was probably not a true sink in the sense of PD mortality exceeding natality. Nevertheless, this reversal in the overall flow of dispersing animals again illustrates the potential for *Y. pestis* to be a “transformer species” in the western U.S. (5).

### Epizootics of Plague as an Adaptation of *Yersinia pestis*?

Epizootics have been identified as a manifestation that “amplifies” *Y. pestis* [e.g., (18, 108)]. The term amplification might imply that epizootics are adaptive, for example by facilitating population growth and expansion of *Y. pestis*. Instead, these epizootic events might be considered as anomalies, triggered by factors that favor PFB cycles. The ecological results of PFB cycles are sometimes destabilizing and can be devastating (109). The explosiveness of PFB epizootics might be a cost of the evolved life history of *Y. pestis* rather than an adaptation; it seems maladaptive for an organism to destroy and sometimes eliminate essential habitat (herein, hosts and fleas).

Perhaps plague epizootic events played little role in the evolution of *Y. pestis*, fleas, and mammalian hosts in Asia where *Y. pestis* originated (110) and these coevolutionary processes had their origins. Plague cycles in Asia are often measured as the prevalence of detected infections in hosts. In populations of great gerbils, plague prevalence is reportedly “always low” (47). In North America, host mortality is pervasive at enzootic and epizootic levels [e.g., (20, 21, 23)]. As an invader in North America, *Y. pestis* may be subject to accidental juxtaposition of conditions favorable to a non-adaptive outcome for all players. Nevertheless, runaway PFB-driven outbreaks might have resulted in evolutionary consequences for *Y. pestis*. For example, periodically destroying its own habitat might have favored mechanisms for *Y. pestis* survival under hostile conditions, such as ability to colonize protozoa or survive in soil, fleas, or elsewhere (1, 35, 36, 111).

### Plague, PFB Cycles, Conservation, and One Health

In this paper, we emphasize the transition of plague activity from enzootic to epizootic explosions due to PFB. Our intent herein has been to focus primarily on the PFB loops that likely occur during an epizootic outbreak of plague, and to propose that those expanding cycles are a central element of epizootics as we narrowly define them (Figure 1). For an epizootic with PFB to occur, there must be adequate (although sometimes relatively low) densities of PDs distributed sufficiently uniformly to allow the rapid expansion of PFB to occur. There also needs to be adequate densities of fleas at the starting point.

The change in flea:host ratios during epizootics have been recognized and repeatedly mentioned for more than 50 years, and the recognition of plague as an enzootic phenomenon (smoldering), as well as exploding into epizootics, is also historic. We suggest these phenomena, coupled with the relatively inefficient transmission of *Y. pestis* by fleas, as pivotal to understanding both the evolution of *Y. pestis* and the ecological manifestations of plague. In PDs at least, the breakdown of territories during epizootics likely contributes substantially as a second reinforcing PFB loop. Our synthesis is a recasting of earlier discussions and observations into a theme that emphasizes sustained transmission and mortality caused by enzootic plague as a common starting point for epizootics, and centering on PFB as the amplifying centerpiece. PFB loops might be initiated by triggers; we speculated on anthropogenic triggers for the plague-flea epizootic loop and provided some supporting evidence.

This reevaluation seemed useful because the history surrounding plague has tended to dampen such thinking. Plague initially received most attention as a series of human epidemics, and public health investigators later recognized epizootic outbreaks of sylvatic plague as elevating the risk to human health (2). The focus on epizootics and epidemics motivated conversations (at least) about how such cycles could be adaptive and diverted attention from thinking about the more common conditions under which natural selection likely molded the life history attributes of *Y. pestis*.

If epizootics are not a necessary component of plague maintenance, and *Y. pestis* evolved a lifestyle that requires high vector loads and high levels of bacteremia to persist (65), we might expect host mortalities to be chronically high even without epizootics, especially in ecosystems where plague is not native. Mammalian species that can persist with sustained high population losses (e.g., PDs) may serve as reservoirs for *Y. pestis*, but plague spillover into associated bystander species, even during enzootic periods, might result in their extirpation or extinction (e.g., as exemplified by black-footed ferrets). There may be mammal communities where epizootics, as we define them, are rare or absent (e.g., due to consistently low flea parasitism or intense territoriality). Lack of noticeable epizootic outbreaks should not be equated with lack of ecological impact of plague.

## Data Availability

All datasets generated for this study are included in the manuscript and/ or the Supplementary Files.

## Author Contributions

DB accumulated notes on this PFB hypothesis over the past decade and collected data on PD shooting in Montana. DE added new ideas on PFB and collected data on PD poisoning in South Dakota. Both authors performed data analyses. DB drafted the original manuscript. DE made additions and substantial organizational revisions. Both authors approved the final version.

### Conflict of Interest Statement

The authors declare that the research was conducted in the absence of any commercial or financial relationships that could be construed as a potential conflict of interest.

## Supplementary Material

Table S1 Fleas Collected after Recreational Shooting on a Prairie Dog Colony in Montana.

Table S2 Fleas Collected Before and After Poisoning on a Prairie Dog Colony in South Dakota.
